# Genome-wide identification and immune response analysis of mitogen-activated protein kinase cascades in tea geometrid, *Ectropis grisescens* Warren (Geometridae, Lepidoptera)

**DOI:** 10.1186/s12864-023-09446-7

**Published:** 2023-06-22

**Authors:** Xiaozhu Wu, Chenghua Zhou, Xiaofang Li, Jingyi Lin, Luis Carlos Ramos Aguila, Feng Wen, Liande Wang

**Affiliations:** 1 State Key Laboratory of Ecological Pest Control for Fujian and Taiwan Crops; Key Laboratory of Biopesticides and Chemical Biology, Ministry of Education, College of Plant Protection, Fujian Agriculture and Forestry University, Fuzhou, 350002 China; 2 School of Pharmacy and Life Science, Jiujiang University, Jiujiang, 332000 China; 3 School of Biological Science and Food Engineering, Chuzhou University, Chuzhou, 239099 China

**Keywords:** *Ectropis grisescens*, *Metarhizium anisopliae*, MAP Kinase, Expression pattern, Signal transduction

## Abstract

**Background:**

Tea geometrid *Ectropis grisescens* (Geometridae: Lepidoptera), is one of the most destructive defoliators in tea plantations in China. The MAPK cascade is known to be an evolutionarily conserved signaling module, acting as pivotal cores of host–pathogen interactions. Although the chromosome-level reference genome of *E. grisescens* was published, the whole MAPK cascade gene family has not been fully identified yet, especially the expression patterns of MAPK cascade gene family members upon an ecological biopesticide, *Metarhizium anisopliae*, remains to be understood.

**Results:**

In this study, we have identified 19 MAPK cascade gene family members in *E. grisescens*, including 5 MAPKs, 4 MAP2Ks, 8 MAP3Ks, and 2 MAP4Ks. The molecular evolution characteristics of the whole Eg-MAPK cascade gene family, including gene structures, protein structural organization, chromosomal localization, orthologs construction and gene duplication, were systematically investigated. Our results showed that the members of Eg-MAPK cascade gene family were unevenly distributed in 13 chromosomes, and the clustered members in each group shared similar structures of the genes and proteins. Gene expression data revealed that *MAPK* cascade genes were expressed in all four developmental stages of *E. grisescens* and were fairly and evenly distributed in four different larva tissues. Importantly, most of the *MAPK* cascade genes were induced or constitutively expressed upon *M. anisopliae* infection.

**Conclusions:**

In summary, the present study was one of few studies on MAPK cascade gene in *E. grisescens*. The characterization and expression profiles of Eg-MAPK cascades genes might help develop new ecofriendly biological insecticides to protect tea trees.

**Supplementary Information:**

The online version contains supplementary material available at 10.1186/s12864-023-09446-7.

## Background

Insects are one of the most speciose group of arthropods on the planet, with approximately 1.5 million species, which contains a large number of agricultural pests consuming 5 to 20% of major grain crops [1, 2]. To survive, insects must continually evolve strategies to resist infection and colonization by pathogenic microbes that would invade and disrupt their tissues [3, 4]. Typically, the mitogen activated protein kinase (MAPK) cascade plays a crucial role in host–pathogen interactions, from pathogen recognition to the triggering of immune responses [5, 6]. MAPKs are serine-threonine protein kinases involved in ancient signal transduction pathways that regulate various cellular processes, including growth and development, metabolism, cell death and immune responses [7–9].

A typical MAPK signaling pathway functions as a multi-tiered phosphorylation signaling cascade composed of MAPK, MAPK kinase (MAP2K/MKK), MAP2K kinase (MAP3K/MEKK) and MAP3K kinase (MAP4K), and participate in multicellular processes [10–12]. In mammals and plants, pathogen-associated molecular patterns (PAMPs) and pattern recognition receptors (PRRs) could activate MAPK signaling pathway and then triggered innate and adaptive immune responses by phosphorylating related downstream components [5, 10, 13–15]. To date, the role of MAPKs in anti-fungal and anti-bacterial pathogens has been extensively studied and a wealth of information has been obtained from many insects and nematodes, including *Caenorhabditis elegans*, *Drosophila melanogaster*, *Anopheles gambiae* and *Plutella xylostella* [16–19]. For example, the Ras/MAPK pathway was required for intrinsic suppression of immune deficiency (IMD) signaling in cultured cells and all immune tissues in the *D. melanogaster*, including hemocytes, fat body and adult intestinal stem cells [17]. Furthermore, Jun N-terminal kinase (JNK) activity is required for Toll-induced cell death by promoting the production of reactive oxygen species (ROS), which participates in the melanization response during wound healing and kills pathogens [20, 21]. Contrarily, treatment of cultured *D. melanogaster* cells with the p38 inhibitor SB203580 enhanced lipopolysaccharide-induced expression of antimicrobial peptide genes, such as Attacin and Cecropin, suggesting that p38 might act as a negative regulator of immune responses [22, 23]. Recent studies carried out by Guo et al.(2020 and 2021) [6, 19] linked the downregulation of ALP and two ABCC toxin receptor genes with an MAPK signaling pathway in a Cry1Ac-resistant strain of *P. xylostella*, and confirmed that the MAPK signaling cascade modulated by insect hormone was involved in the differential expression of diverse midgut genes to cope with the insecticidal action of Cry1Ac toxin.

Studies of host–pathogen interactions (HPI) have provided valuable insights into the highly aggressive coevolution between entomopathogenic fungi (EPFs) and their insect hosts, as well as improving the invasiveness and toxicity of EPFs to insects. A numerous reports have demonstrated that insect hosts can develop resistance to biological control agents (BCAs), such as fungi and bacteria, in the same way that they developed resistance to chemical pesticides [24, 25]. At least 27 species of insects have developed resistance to the most used bio-pesticide in the world, *Bacillus thuringiensis* [25–27]. For instance, Dubovskiy et al*.* (2013) [24] reported that the larvae of the 25^th^ generation of the Greater wax moth, *Galleria mellonella*, showed significantly enhanced resistance under constant selective pressure from the insect pathogenic fungus *Beauveria bassiana*. In another study, compared to unselected control lines, *D. melanogaster* larvae from the fungal-selected lines showed no increase in resistance, but had higher survival rates in the presence of *Aspergillus nidulans*, and exhibited a reduced sensitivity to sterigmatocystin, a toxin produced by this fungus. However, the underlying mechanism for the increased toxin and pathogen specific tolerance is unclear [28]. A number of studies revealed MAP kinases such as JNK and p38 were activated by pathogens in insect cells, and appeared to mediate pathogen defense-related gene expression [23, 29, 30]. For example, MKK4-JNK pathway in response to microbial challenge is essential for the release of normal antimicrobial peptides, and the activation of this pathway triggered the translocation of transcription factors, such as activator protein-1 (AP-1) proteins to the nucleus, contributing to the production of immune effectors, such as antimicrobial peptides (AMPs) [31, 32]. Further, it has been well proved that the TAK1/JNK/AP-1 signaling pathway and NF-kB signaling are both required to activate antimicrobial peptide gene expression during the immune response in the *D. melanogaster* fat body [33].

Tea is one of the most important and non-alcohol beverages with economic significance globally, which is now grown in almost 60 countries [34–36]. *Ectropis grisescens* (Geometridae, Lepidoptera), also called the tea geometrid, is one of the most destructive leaf-eating pest insects in tea plantations in China, causing significant losses to tea crops in terms of yield and quality [37–40]. For many decades, spraying chemical pesticides was the most efficient way to control outbreaks of tea geometrids, which might cause food safety problems and environmental issues [41, 42]. Biocontrol was one of the most effective alternatives, in particularly, biological plant protection with EPFs played a key role in sustainable pest management program, because of its advantages, including low costs, high efficiency, safety for beneficial organisms, and reduction of environmental residues [43–45]. For example, *Metarhizium anisopliae* and *B. bassiana* were the most widely used EPFs and develop as type of ecological biopesticides [46, 47]. Although the differences of immune gene expression levels in fat bodies and hemocytes of *Ectropis obliqua* have been detected after being infected by the EPFs, the molecular mechanisms of tea geometrid defenses against EPFs were still unclear [48].

In this study, we have systematically identified all MAPK cascade gene family members from *E. grisescens* genome and their evolutionary relationship in terms of phylogenetic analysis, chromosomal localization and gene duplication with other arthropods. The evolutionary analysis presented the conservation of the Eg-MAPK cascade and, for the first time, identifies the evolutionary origin of the complete set of *Eg-*MAPK cascade genes. The expression patterns demonstrate differing roles for *Eg-MAPK* cascade genes in response to EPFs, suggesting a conserved MAPK architecture for immune signal transduction pathway. Our characterization and expression analysis of *Eg-MAPK* cascade genes lays the foundation for further investigation on the functions of MAPK cascade genes in entomopathogen resistance, might point the way to develop biological insecticides to better control this pest.

## Results and discussion

### Identification of Eg-MAPK cascade genes from the E. grisescens genome

**Fig. 1:**
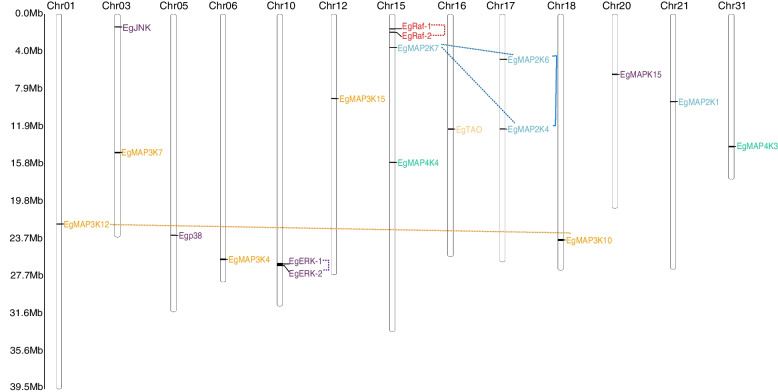
Chromosome distribution of *MAPK* cascade genes in *E. grisescens*. The chromosome numbers are indicated at the top of each chromosome image. Gene duplication analysis of *Eg-MAPK* cascade genes was also presented with dot lines

**Fig. 2:**
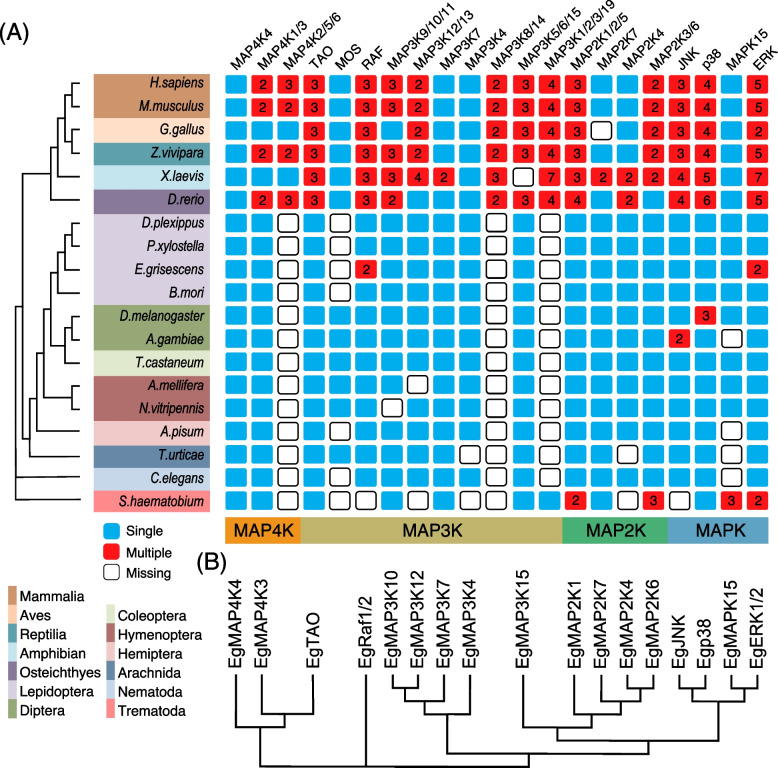
Distribution and evolutionary relationship of MAPK cascade genes. **A** Distribution of MAPK cascade genes among 19 animal species, including 6 vertebrate and 13 invertebrate species. **B** Unrooted phylogenetic relationships among the Eg-MAPK cascade genes

A total of 19 MAPK cascade genes were identified in *E. grisescens*, using *Homo sapiens*, *D. melanogaster* and *P. xylostella* (a Lepidoptera pest with the MAPK cascade genes have been identified) MAPK cascade genes as query sequences (Table 1). The predicted Eg-MAPK cascade genes were named according to the homologs in *D. melanogaster* and *P. xylostella*. The detailed gene information for these *Eg-MAPK* cascade genes, such as gene names, gene locations, peptide lengths, conserved protein kinase domain, and parameters for the deduced polypeptides, are listed in Table 1. Eg-MAPK cascade genes varied markedly from 270 amino acids (EgERK-2) to 1768 amino acids (EgMAP3K4), ranged in molecular mass from 30.6 kDa to 200.3 kDa, and the predicted isoelectric points varied from 5.37 (EgMAP3K7) to 9.25 (EgMAP3K4), which were comparable with MAPKs from other invertebrates species [16–18]. As shown in Table 1, the phosphorylated sites prediction results showed that almost all Eg-MAPK cascade genes, except EgTAO, contained more than two phosphorylated sites, including serine and threonine residue, which was consistent with the fact that MAPKs were a class of important signal transducing serine/threonine-specific protein kinases in cells [49, 50]. The prediction of subcellular localization revealed that most of Eg-MAPK cascade genes were located in the nucleus, cytoplasmic and plasma membrane, demonstrating that the MAPK of such pathways were the molecular link between the plasma membrane sensors and the nuclear transcription factors [51]. The 19 identified *Eg-MAPK* cascade genes were unevenly distributed on 13 of the 31 chromosomes of *E. grisescens*, in which, chromosome 15 contained the most *Eg-MAPK* cascade genes (Fig. 1). From the distribution of MAPK cascade orthologs among other arthropods and model animals, it was obvious that the invertebrate species had fewer MAPK cascade genes than the vertebrate species (Fig. 2A), which was consistent with the precious reports that vertebrates had significantly more MAPK family members than invertebrates [52]. Given that MAPK and MAPKK have much fewer members than MAPKKKs; this suggested that the MAPK and MAPKK genes tend to be more conserved in evolution than MAPKKKs, showing incredible consistency in both animals and plants [53, 54]. To explore the evolutionary relationships of Eg-MAPK cascade genes, all *Eg-MAPK* cascade genes full-length coding sequences (CDS) were used to construct an unrooted tree (Fig. 2B). Based upon sequence homology, Eg-MAPK cascade genes were clustered into four groups (MAP4K, MAP3K, MAP2K and MAPK), were consistent with the previous works in *C. elegans*, *D. melanogaster*, *A. gambiae* and *P. xylostella* [6, 16–18]. However, *EgTAO* and *EgMAP3K15* did not cluster together with other MAP3K members and formed a single clade, indicating that evident gene expansion or divergence occurred in the MAP3K group of *E. grisescens* during evolution. Furthermore, tea geometrid lacked the *mos* gene like other Lepidoptera species but contained dual *ERK* and *Raf* genes.

**Table 1:** Characterization of *MAPK* cascade genes in *E. grisescens*

**Gene name**	**Chr**	**ORF (bp)**	**Exon No**	**Pepide length**	**PKD**	**PI**	**MW (kD)**	**Predict phosphorylation site (Exp.&string)**	**Subcellular localization**^a^
EgERK-2	chr10	813	4	270	3–194	6.85	30.6	S(85, 196); T(68, 73, 186, 199, 204, 210, 254)	mito: 13, nucl: 9.5, cyto: 9, extr: 4
EgERK-1	chr10	1095	8	364	28–316	5.87	42.0	S(6, 205, 314); T(97, 188,193,306)	cysk: 19, cyto: 9, pero: 5.8, nucl: 5.3
EgMAPK15	chr20	3141	23	1046	40–336	8.51	120.3	S(2, 262, 391, 393, 420, 432)	nucl: 20, cyto: 11
Egp38	chr05	1083	9	360	20–304	5.93	41.6	S(250, 316); T(24, 176, 181)	cyto: 15, mito: 8, nucl: 6, pero: 2
EgJNK	chr03	1287	9	428	68–364	7.01	48.7	S(24, 34, 335); T(22, 220, 225, 230, 428)	mito: 23, pero: 3, cyto: 2.5, nucl: 2.5
EgMAP2K6	chr17	1008	3	335	51–310	6.66	37.9	S(2, 158, 198,204, 335); T(32, 81, 208, 277)	nucl: 13, cyto: 12, mito: 7
EgMAP2K4	chr17	1233	8	410	109–370	8.85	46.3	S(2, 80, 104, 130, 140, 262, 397,); T(103, 266, 338, 401)	cyto: 13.5, nucl: 8.5, pero: 5, mito: 4
EgMAP2K7	chr15	1668	13	555	96–354	9.17	61.0	S(2, 4, 49, 60, 245, 383, 439, 461, 471, 479, 515, 517, 521, 525); T(64, 249, 387, 455)	cyto: 15.5, nucl: 12.5, golg: 2
EgMAP2K1	chr21	1296	9	431	89–368	8.63	47.6	S(2, 5, 34, 110, 185, 239, 243, 252, 304, 311, 429); T(25, 29, 36, 38, 49, 247)	cyto: 18, mito: 13
EgMAP3K15	chr12	4122	25	1373	625–882	5.78	155.5	S(217, 351, 408, 467, 707, 930, 953, 989, 990, 995, 1029, 1103,1145, 1177, 1238, 1319, 1320, 1342); T(95, 173, 723, 786, 872, 943, 971, 1027, 1172, 1178, 1221, 1313, 1347, 1350)	nucl: 16, cyto: 14
EgMAP3K4	chr06	5307	29	1768	1563–1750	9.25	200.3	S(99, 114, 137, 140, 161, 168, 216, 279, 294, 299, 337, 393, 405, 415, 441, 487, 529, 533, 668, 1092, 1137, 1188, 1202, 1362); T(360, 434, 537, 541, 552, 555, 621, 817, 939, 1095, 1200, 1364, 1452, 1645, 1656, 1748)	nucl: 21, cyto: 7, plas: 2, golg: 2
EgMAP3K7	chr03	2640	16	879	223–427	5.37	97.6	S(379, 511, 578, 630, 659, 758, 777, 790); T(58, 156, 371, 374, 537, 634, 678)	cyto: 13, nucl: 11, plas: 3, pero: 3, extr: 2
EgMAP3K12	chr01	1458	8	485	32–273	6.87	54.7	S(313); T(180)	cyto: 13, nucl: 7, pero: 5, mito: 4, extr: 3
EgMAP3K10	chr18	3249	11	1082	96–372	8.36	121.7	S(261, 507, 545, 853, 1047, 1066); T(63, 246, 257, 265, 838, 851, 1082)	nucl: 18.5, cyto: 8.5, plas: 4
EgRaf-1	chr15	2211	12	736	412–676	8.91	83.9	S(22, 31, 77, 103, 111, 312, 342, 345, 346, 372, 386, 478, 687); T(30, 369, 443)	plas: 13, nucl: 8.5, cyto: 6.5
EgRaf-2	chr15	2214	12	737	413–677	8.91	84.0	S(22, 31, 77, 103, 111, 313, 343, 346, 347, 373, 387, 479, 688); T(30, 370, 444)	plas: 13, nucl: 8.5, cyto: 6.5
EgTAO	chr16	3153	21	1050	86–339	8.48	121.8	NA	nucl: 24, cyto: 4, plas: 3
EgMAP4K3	chr31	3366	22	1121	21–275	8.57	125.7	T(516, 720, 747)	nucl: 15.5, cyto: 11, pero: 3
EgMAP4K4	chr15	4041	22	1346	28–292	8.97	149.2	S(12, 145, 232, 384, 430, 431, 434, 436, 437, 439, 537, 560, 608, 702, 717, 828, 831, 835, 837, 924, 968, 972, 977, 1005, 1009, 1010, 1013, 1026, 1119, 1290); T(62, 123, 165, 190, 194, 357, 427, 454, 462, 472, 580, 667, 683, 684, 758, 794, 877, 938, 946, 993, 1071)	nucl: 22.5, cyto: 9.5

^a^*cysk* Cytoskeleton, *cyto* Cytoplasmic, *extr* Extracellular, *golg* Golgi complex, *mito* Mitochondrial, *nucl* Nuclear, *pero* Peroxysome, *plas* Plasma membrane

### Structural divergence of Eg-MAPK cascade genes

**Fig. 3:**
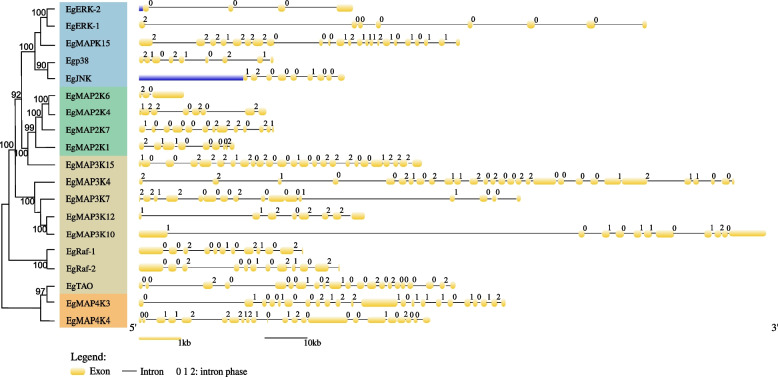
Gene structures of *Eg-MAPK* cascade genes. The exons and introns are represented by boxes and lines, respectively. Number 0, 1 and 2 indicated the intron splicing phase 0 (located between two codons), phase 1 (splitting codons between the first and second nucleotides) or phase 2 (splitting codons between the second and third nucleotides), respectively

Gene structure divergence plays an essential role in the evolution of gene families and provides additional information to evaluate possible structural evolutionary relationships among gene family members. Non-coding sequences, such as introns, were regarded as an indicator of genome complexity, providing insights into genome evolution. Therefore, the gene structure, exon position and phases of intron in *Eg-MAPK* cascade genes were analyzed through comparing their coding sequence and genomic sequence by GSGD online server [55]. In general, the numbers of introns and exons were highly variable in *Eg-MAPK* cascade genes, even in the same group (*MAP4K*, *MAP3K*, *MAP2K* and *MAPK*), and ranged from 3 to 28, suggesting that the *MAPK* cascade genes might have originated in different ancestors (Fig. 3). For example, MAP2K genes showed significant differences in the number of exons and introns (Fig. 3). The replication events might be likely to have occurred in ancient times, and the descendant genes evolved into diverse exon–intron structures to perform different functions in the *E. grisescens* genome [56]. In addition, the MAPK genes belonging to the same clade had similar gene structure, for example, EgJNK and Egp38 shared similar exon–intron structure. A certain degree of conservation could be also observed in the Eg-MAP3K genes. For instance, the paralogous gene pairs generally showed highly similar gene structure, such as *EgMAP3K10*/*EgMAP3K12*, and *EgRaf-1*/*EgRaf-2*, suggesting that these MAPK paralogous gene might be derived from the same ancestral gene, and might have functional redundancy [57].

**Fig. 4:**
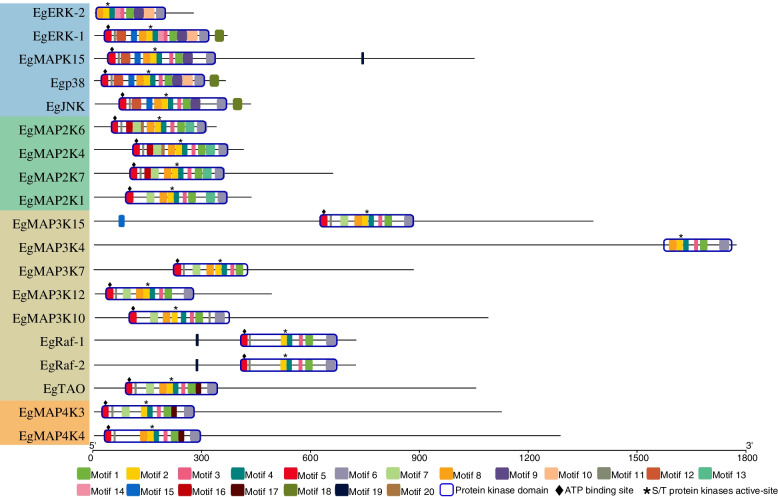
Protein structures of *Eg-MAPK* cascade gene family members. Different motif is represented by specific color. Blue hollow box indicated the protein kinase domain (IPR000719), black diamond indicated the ATP binding site (IPR017441) and the asterisk indicated serine/threonine-protein kinase active site (IPR008271)

**Fig. 5:**
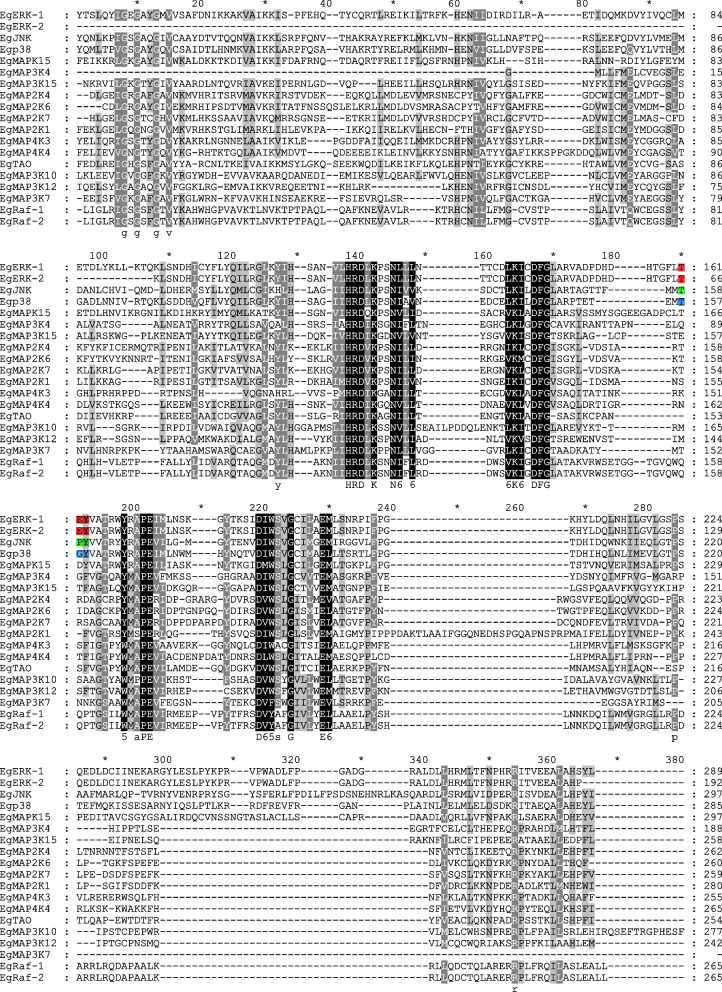
ClustalW amino acid sequence alignment of typical protein kinase domain in *Eg-MAPK* cascade proteins. Gaps (dots) have been inserted for optimal alignment. Black and light gray shading indicate the presence of identical and conversed amino acid residues, respectively. Consensus amino acid residues are shown below the alignment. The motif TEY, TPY and TGY have been highlighted by red, green and blue color, respectively

Further, motif and domain analyses were performed based on amino acid sequences, which can help us investigate their function. Generally, 20 conserved motifs within Eg-MAPK cascade gene family members were identified using online MEME tools (Fig. 4). Although the gene structures were highly variable, we noted that all Eg-MAPK cascade gene family members contained a series of relatively conserved motifs. Four of the motifs (motif 1, 2, 3 and 4) were shared by all Eg-MAPK cascade gene family members. Almost all Eg-MAPK cascade gene family members contained motif 5, except EgERK-2 and EgMAP3K4. Similarly, only EgMAP3K7 did not have motif 6. Meanwhile, the conserved domain structures revealed similar motifs among each group. The motif analysis results illustrated that conserved motif structures within groups supported close evolutionary relationships, and there might be functional divergences among different groups. For example, motif 12 and 18 was only present in EgERK-1, Egp38 and EgJNK, while most of this group members did not contain motif 7. Interestingly, motif 13 appeared to be distinctive in EgMAPKK group, implying that this motif might perform unique functions in the physiological behavior of EgMAPKK group members. Further, the results showed that MAP3K exhibits higher diversity, not only in gene structure (Fig. 3) but also in their protein sequences (Fig. 4), when compared with MAP4K, MAP2K and MAPK. For example, EgMAP3K15 showed obviously different among other MAP3Ks, which contained motif 15 at the N-terminal of the protein sequence, while EgMAP3K7 was lack of motif 6 at the C-terminal of the protein kinase domain (PKD). Subsequently, the protein structure of Eg-MAPK cascade gene family members was predicted using InterPro to analyze the conserved domains, following a conserved protein kinase domain sequence alignment analysis by clustalW (Figs. 4 and 5). Results showed that all Eg-MAPK cascade gene family members identified in our study possessed the PKD (IPR000719) flanked by N- and C-terminal regions of different lengths, which was considered to be the typical MAPK structure of phosphating downstream proteins [58]. Compared with the results of MEME analysis, it can be found that the PKD of Eg-MAPK cascade gene family members mainly consisted of motifs 1–11 as shown in Fig. 4. PKD contained motif 5 and 2 as ATP binding site (IPR017441) and serine/threonine-protein kinase active site (IPR008271), respectively. These results revealed that Eg-MAPK cascade gene family members could use ATP as a phosphate base source for target phosphorylation by transferring the ATP’s gamma phosphate to amino acid residues (such as serine and threonine) in the MAPK cascade signal transduction pathways [59]. MAPKs themselves were reported to be phosphorylated at residues in a region known as the activation loop, where two key residues, a threonine and a tyrosine residue, were separated by a single amino acid (TXY motif) [59]. Similar to other species, EgERK1/2 contained the motif Thr-Glu-Tyr in its activation loop, whereas EgJNK and Egp38 contained Thr-Pro-Tyr and Thr-Gly-Tyr, respectively (Fig. 5) [60, 61]. The alignment results showed that the PKD residue sequences were conserved within the group members and diverged relatively between the groups (Fig. 5).

### Phylogenetics and synteny analysis of Eg-MAPK cascade genes

**Fig. 6:**
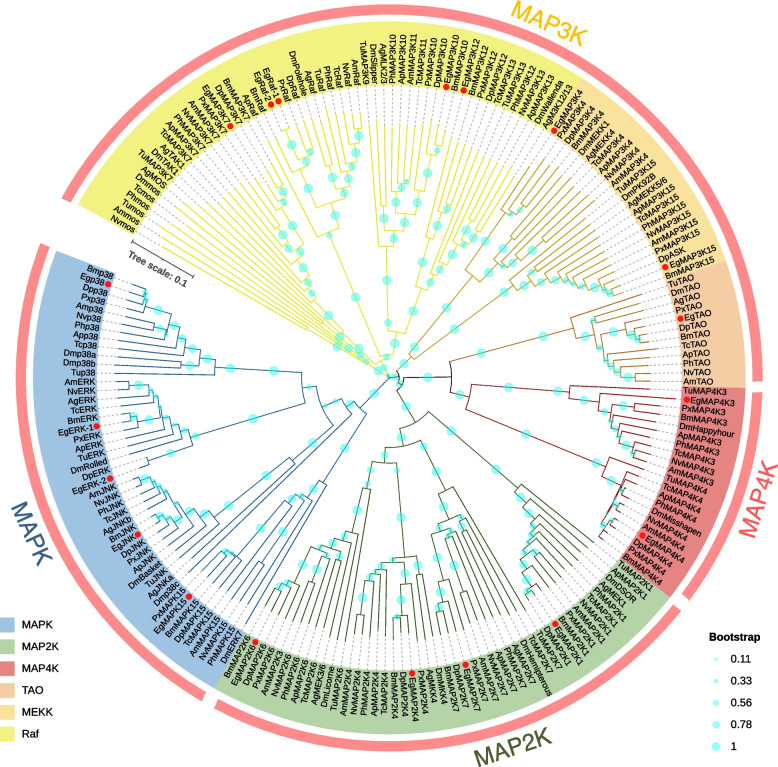
Neighbor-joining analyses of MAPK, MAP2K, MAP3K and MAP4K from *E. grisescens* and 11 other arthropod species. MAP3Ks include three clades: Raf, MEKK and TAO. The solid red circle represents Eg-MAPK cascade gene members

**Fig. 7:**
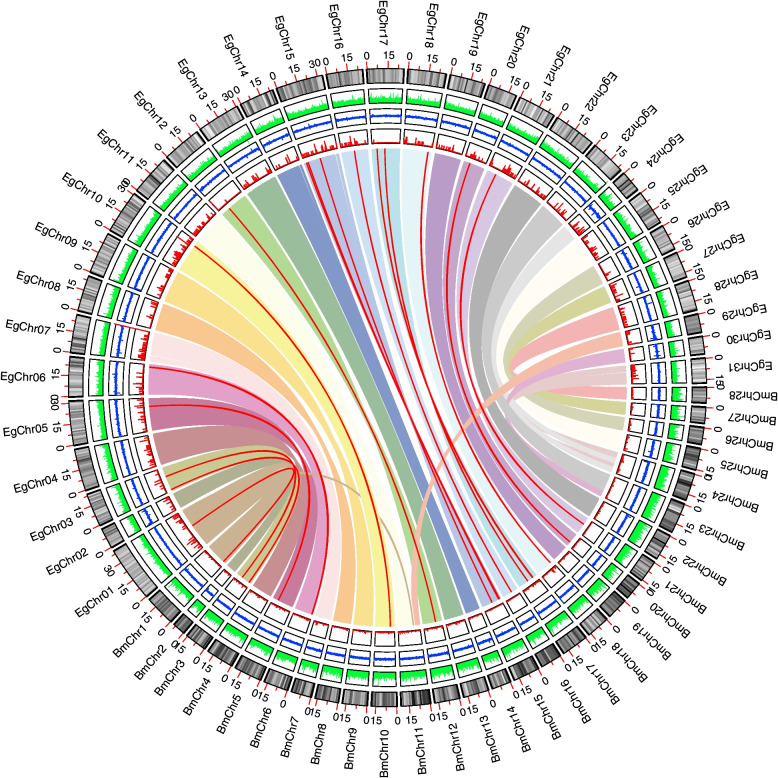
Synteny analysis of *MAPK* cascade genes between *E. grisescens* and *B. mori*. Coloured arcs indicate homologous genomic blocks between *E. grisescens* and *B. mori*, while the red lines highlight the syntenic *MAPK* cascade genes pairs. Schematic representation was displayed by using the CIRCOS software. The size of chromosomes was consistent with the actual pseudo-chromosome size. Positions are in Mb

To explore the function and evolutionary relationships of MAPK cascade genes between *E. grisescens* and other arthropod species, phylogenetic trees were constructed from alignments of complete PKD sequences of MAPK cascade genes using the Neighbor-Joining (NJ) method by MEGA-X. The NJ phylogenetic distribution indicated that the organization of MAPK cascade genes could be divided into six groups including MAPK, MAP2K, MEKK, TAO, Raf and MAP4K. In the MAPK cascades, MAP3K exhibited higher diversity compared with MAP4K, MAP2K and MAPK, which was divided into three major clades. The Raf clade was composed of MAP3K7, MAP3K10/11, MAP3K12/13, mos and Raf members, while the MEKK clade contained MAP3K4 and MAP3K15 (Fig. 6). TAOs functioned as MAP3Ks in MAPK cascades which doubly phosphorylated and activated the MAP kinase kinases (MAP2Ks) MEK3 and MEK6 [62, 63], but the sequence of TAO was more closely related to MAP4K in the phylogenetic tree (Fig. 6), which was consistent with previous reports [6, 64]. All arthropod species contained four MAP2Ks (MAP2K1, MAP2K3/6, MAP2K4 and MAP2K7), and two MAP4Ks (MAP4K3 and MAP4K4), with each MAP2K and MAP4K members clustered conservatively in a single clade. The MAPK group contained four types of MAPK (ERK, JNK, p38 and MAPK15), among which the protein sequence similarity between MAPK15 and other MAPKs was low, suggesting that MAPK15 may not be a classical MAPK. The phylogenetic similarity found in *E. grisescens* and other species in family Lepidoptera, suggesting that they may have evolved conservatively, which was consistent with the synteny analysis results between *E. grisescens* and *B. mori* (Fig. 7). Furthermore, we compared the genomic structures of *E. grisescens* with the model species of Lepidoptera, *B. mori*. The results showed that 8687 collinear gene pairs were found between *E. grisescens* and *B. mori* genome, indicating that a large number of syntenic relationship events existed between *E. grisescens* and *B. mori*. Among these collinear genes, a result of 18 collinear MAPK cascade gene pairs demonstrated that many consensuses in MAPK cascade gene may have existed before the species divergence between *E. grisescens* and *B. mori*, implying that MAPK cascade was an ancient and highly evolutionarily conserved signaling pathway [65–67].

### Spatial and temporal expression profiles of Eg-MAPK cascade genes

**Fig. 8:**
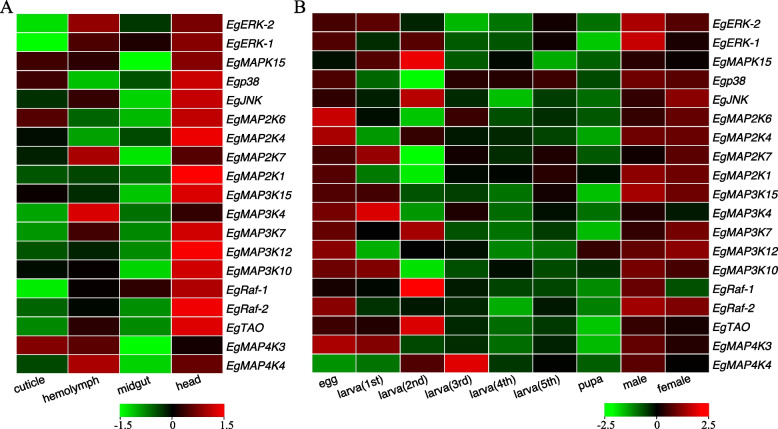
Spatial (**A**) and temporal (**B**) expression profiles of *Eg-MAPK* cascade genes. The expression profile was shown by a green–red gradient

Expression profiling of gene families was the determination of the pattern of genes transcriptional level, which could give a global picture of cellular function of gene families [68]. Previous findings have shown that MAPK signaling pathways were activated in response to various extracellular factors, resulting in transcriptional activation of immediate early genes that influenced many tissue and stage-specific biological activities, as cell proliferation, survival and differentiation, which were essential for insect growth and organ development, such as oocyte maturation, pupariation, eclosion, wing growth, etc. [69–72]. For instance, ERK pathway possibly regulated ecdysone biosynthesis, while p38 pathway might be involved in the germline stem cell development and differentiation in the cabbage beetle [72]. Since no *E. grisescens MAPK* cascade genes have been previously documented, and to investigate the potential functions of *MAPK* cascade genes in *E. grisescens* growth and organ development, we analyzed the expression patterns of *Eg-MAPK* cascade genes in different tissues and stages of *E. grisescens*. The result showed that 19 *MAPK* cascade genes could be detected in all tissues/organs of fifth instar larvae according to the expression levels (Fig. 8A). Head had the highest number (14) of highest-level expressed genes among the detected tissues or organs, while the hemolymph had four genes, which was similar with the previous result in *P. xylostella* [6]. However, the expression level of *MAPK* cascade genes was extremely low in the midgut. In general, *MAPK* cascade genes were expressed in all four developmental stages (egg, larva, pupa and adult) of *E. grisescens*. The expression profile indicated that most of *MAPK* cascade genes were highly expressed in egg, first-instar larva and adult stages, suggesting that they might be involved in the physiological processes of organ and embryonic development (Fig. 8B) [73]. Similarly, RNA-seq analysis of *MAPK* cascade genes in *P. xylostella* showed that most of the MAPK cascade genes had high expression levels in both eggs and adults, which was consistent with the expression profiles of *Eg-MAPK* cascade genes in this study [6]. These data indicate that the *MAPK* cascade genes might be involved not only in the organ growth and development, but also in embryo development.

### Expression profile of Eg-MAPK cascade genes after infection with M. anisopliae

**Fig. 9:**
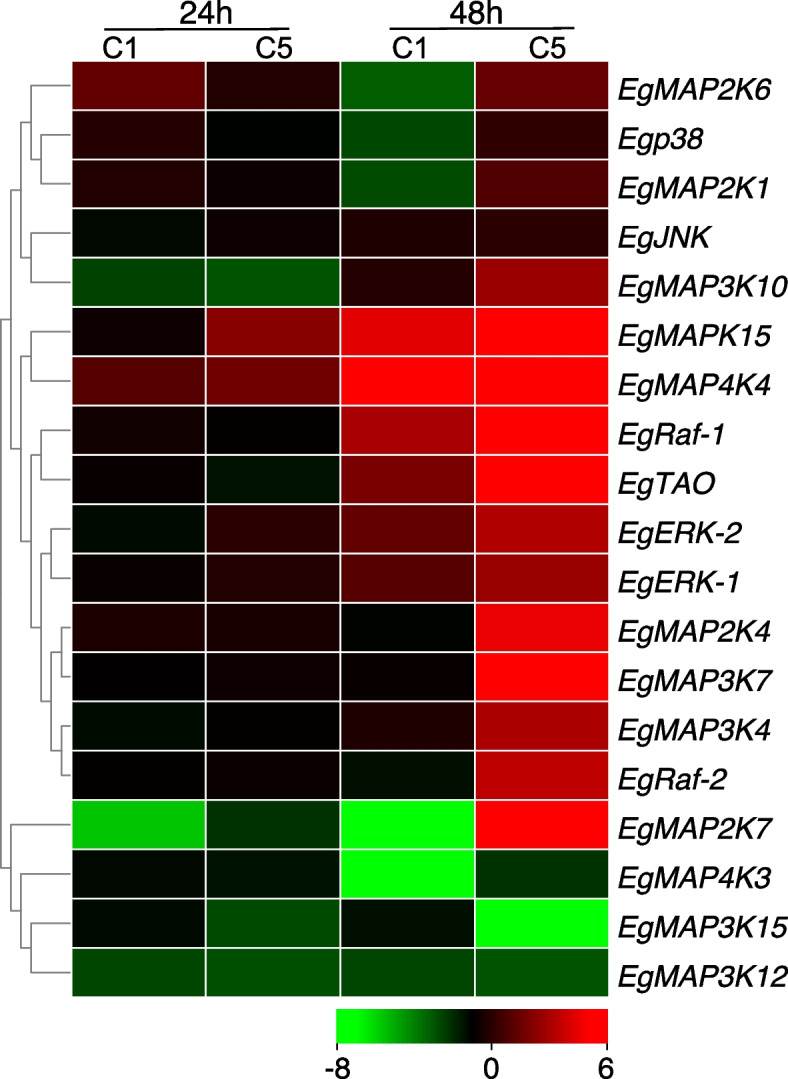
Expression patterns of *Eg-MAPK* cascade genes in response to *M. anisopliae* infection. Levels of down expression (green) or up expression (red) are shown on a log2 scale from the high to the low expression of each *Eg-MAPK* cascade genes. C1 and C5 indicated the treatment of 1 × and 5 × 10^7^ mL^−1^ M*. anisopliae* conidial suspension, respectively

Accumulating evidence suggested that many insect MAPK cascade genes had been considered to be involved in the innate immune response against pathogens. For example, the function of the D-p38 MAP kinases regulating insect immunity was to reduce the expression of antimicrobial peptide gene after exposure to lipopolysaccharide [23]. Guo et al*.* (2021) [6] reported that the transcript levels of most of the MAPK cascade genes in *P. xylostella* were up-regulated in the midgut tissues of all Cry1Ac resistant strains compared to the susceptible strain, as well as the protein expression and the phosphorylation levels, suggesting that MAPK signaling cascade played as a general regulator in the diamondback moth to cope with the entomopathogenic bacterium *B. thuringiensis*. To discover the Eg-MAPK cascade genes that are involved in immune response, the changes in the expression level of *Eg-MAPK* cascade genes were analyzed after infection with *M. anisopliae*, a widely used EPF. We found that eleven *Eg-MAPK* cascade genes were significantly (*p* < 0.05) up-regulated or down-regulated with at least 2 folds in treatment group injected with *M. anisopliae* conidial suspension (2 µL, 5 × 10^7^ conidia mL^−1^) after 48 h compared to the controls (2 µL, Tween 80 solution). In general, *Eg-MAPK* cascade genes were strongly up-regulated 48 h after infection with *M. anisopliae* compared with 24 h (Fig. 9). *EgMAPK15*, *EgRaf-1* and *EgMAP4K4* were significantly up-regulated in conidial suspension concentrations (1 × 10^7^ and 5 × 10^7^ conidia mL^−1^) 48 h after infection, indicating that these genes might contribute important function in response to EPFs infection. *EgMAP3K12*, *EgMAP3K15* and *EgMAP4K3* were strongly down-regulated infection with *M. anisopliae*, suggesting these genes might act as negative regulators in immune response against pathogens (Fig. 9). Interestingly, the expression patterns of dual *EgERK* (*ERK-1* and *ERK-2*) and *EgRaf* (*Raf-1* and *Raf-2*) were similar after infection with *M. anisopliae*, as well as in the spatial and temporal expression profiles, suggesting these dual *ERK* and *Raf* genes might be functionally redundant. These results also implied conservation of gene function in the immune response during the evolution of MAPK cascade pathway; however, the function of MAPKs in the regulation of insect immunity remained to be studied in the future.

### Prediction of interaction networks of Eg-MAPK cascade genes

**Fig. 10:**
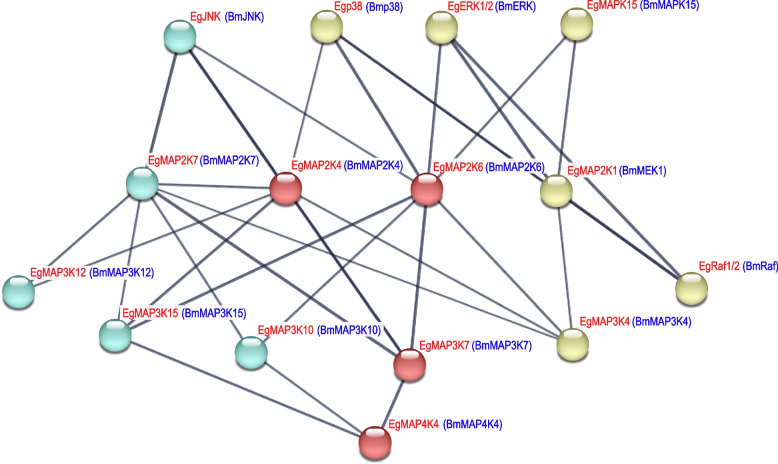
Protein–Protein interaction of *Eg-MAPK* cascade proteins based on BmMAPK orthologs as predicted by STRING search tool. The thickness of the lines represents the level of interaction between proteins

In general, each MAPK cascade consisted of at least three-tier conserved protein kinase (MAP3Ks, MAP2Ks and MAPKs) [74, 75]. In this study, we constructed the predicted interaction network containing 17 Eg-MAPK cascade genes based on *B. mori* homologous genes using STRING 11.0 software with the confidence parameter set at a threshold of 0.7 (Fig. 10). Among these protein kinases, 17 Eg-MAPK cascade genes formed a four-tier interaction network (MAP4K, MAP3Ks, MAP2Ks and MAPKs). For example, EgMAP4K4 was predicted to interact with three EgMAP3Ks, including EgMAP3K7, EgMAP3K10 and EgMAP3K15, which was consistent with the previous result that MAP4K4 could activate TNFα-induced JNK signaling transmission through the kinases TAK1, MKK4 and MKK7 [76]. Similarly, the Raf/MEK/ERK pathway has different effects on growth, prevention of apoptosis, cell cycle arrest and induction of drug resistance in cells of various lineages in human, which might also play critical roles in *E. grisescens* [77, 78]. Furthermore, the interaction network among EgMAP3K4-EgMAP2K1/EgMAP2K6-EgERK1/2 cascade and EgMAP2K7-EgJNK cascade enriched significantly GO term in immune response, including immune response (GO:0006955) and immune system process (GO:0002376) (Additional Table 1). The diagrammatic predicted PPI network could reveal a deductive signaling pathway of Eg-MAPK cascade genes, which provided helpful information for further investigation of the functions of Eg-MAPK cascade genes. However, the accurate regulatory mechanisms among Eg-MAPK cascade genes in immune response required further investigation.

## Conclusions

In summary, we identified 19 *E. grisescens* MAP kinases and systematically analyzed gene characterizations and phylogenies, as well as expression profiles, to provide a basis to explore the functions of MAPKs in immunity signaling pathways in *E. grisescens* in response to EPFs. Our study provides systematical study of MAPK signaling cascades in *E. grisescens*, which were important for signal transduction in entomopathogen resistance, might point the way to develop biological insecticides to better control this pest.

## Methods

### Identification and characterization analysis of Eg-MAPKs

All the *E. grisescens* chromosome-level genome sequence data were obtained from Genbank (Access No. PRJNA660825). The MAPK orthologs of *H. sapiens*, *D. melanogaster* and *P. xylostella* were retrieved from GenBank database (http://www.ncbi.nlm.nih.gov) [6, 12]. To identify the MAPK cascade genes in *E. grisescens*, the *H. sapiens*, *D. melanogaster* and *P. xylostella* MAPK sequences were used as queries to perform a BLASTP search against 18,332 sequences of the protein database of *E. grisescens* with a cutoff e-value ≤ e^−10^. The identified MAPK sequences were further screened by manual editing to remove the redundancy. To verify the reliability of the results, all putative non-redundant MAPKs were assessed with UniProt (http://www.uniprot.org/) and SMART (http://smart.embl-heidelberg.de/) online database, respectively. The theoretical isoelectric point and molecular weight were estimated by pI/Mw tool (http://web.expasy.org/compute_pi), while WoLF PSORT predictor was used to predict the subcellular localization of Eg-MAPK cascade genes (https://wolfpsort.hgc.jp). The conserved protein kinase domains (PKD) of MAPKs were investigated by multiple alignment analyses using ClustalW, and the phylogenetic analysis for Eg-MAPK cascade genes and 11 other arthropod species MAPK cascade genes was performed by using MEGA-X program by the neighbor-joining method, with bootstrap value from 1,000 replicates indicated at each node with the following parameters: p-distance and pairwise deletion.

### Gene structure and chromosomal locations

The gene structure of *Eg-MAPK* cascade genes was displayed by comparing the coding sequences and corresponding genomic DNA sequences with the Gene Structure Display Server tools (http://gsds.cbi.pku.edu.cn/) [55]. The chromosomal locations of the *Eg-MAPK* cascade genes were determined according to the structure annotation file of the *E. grisescens* genome, and mapped by using a TBtools toolkit [79]. The Multiple Collinearity Scan toolkit (MCScanX) was used for the synteny analysis, and the result is graphic by Circos software (http://circos.ca/) [80, 81].

### Protein structure and conserved motif analysis

The MEME program (http://meme-suite.org/) was used to identify the conserved motifs of the Eg-MAPK cascade genes with the following parameters: any number of repetitions of a single motif, the maximum numbers of different motifs up to 20 motifs, the minimum motif width with 6 amino acids, the maximum motif width of a motif with 80 amino acids [82]. Interpro database (http://www.ebi.ac.uk/interpro) was used to identify conserved domains and important sites in Eg-MAPK cascade genes [83]. Subsequently, the TBtools toolkit was used to draw the diagram [79].

### Insect rearing and expression analysis

*E. grisescens* moths were acquired from the Tea Research Institute, Chinese Academy of Agricultural Sciences, Hangzhou, China. The larvae were reared on tea leaves at 23 ± 2 °C and 70–80% relative humidity with a 16 h light/8 h dark photoperiod in the insect-rearing laboratory. The larvae of *E. grisescens* were used for tissue-specific expression analysis and infection treatments according to previous work with some modifications [48]. For tissue-specific expression analysis, fourth-instar larvae were used to collect the head, cuticula, midgut and hemolymph. Eggs, 1^st^ to 5^th^ instar larvae, pupae and adults were collected to determinate the expression patterns of MAPK cascade genes related to growth and development. For infection treatments, fourth-instar larvae were randomly selected and were injected with *M. anisopliae* conidial suspension (2 µL, 1 × and 5 × 10^7^ conidia mL^−1^) and Tween-80 solution (2 µL) using microliter syringes (Shanghai GaoGe Co., Shanghai, China). Samples were stored at − 80 °C after liquid nitrogen freezing if not immediately used for RNA isolation and subsequent analysis. Total RNA from 0.1 g per sample was extracted by the TRIzol method and treated with DNaseI to eliminate any DNA contamination. First-strand cDNA was synthesized according to the instructions for the Hifair® II 1st Strand cDNA Synthesis SuperMix (Yeasen Biotechnology Co., Ltd., Shanghai, China). The expression profiles of *Eg-MAPK* cascade genes were evaluated upon the qPCR analysis using *EgActin* gene as an internal reference gene, and three biological replicates were performed for each experiment. For spatial and temporal expression pattern analysis, the average of total Δ*C*Tvalue (Δ*C*T. average) was subtracted from all other Δ*C*T values to obtain second normal standardization, according to the previous method, using the formula: u = (Δ*C*T–Δ*C*T. average)/σ (in which, u is the value after normal standardization, and σ is the standard deviation) [79, 84]. For infection treatments analysis, the expression levels were calculated from the –ΔΔ*C*T value [–ΔΔ*C*T = (*C*Tcontrol.gene – *C*Tcontrol.actin) – (*C*Ttreat.gene – *C*Ttreat.actin)], and a heatmap was generated by TBtools toolkit. Two tailed Student's t-test (P_0.05_) was used to determine the significant difference of relative expression of individual *Eg-MAPK* cascade genes between control and different treatments (Microsoft Excel 2007). Fold-change greater than 2 with *p-value* of < 0.05 was defined as up-regulated gene, while a fold change of 0.5 or less was used to define down-regulated genes when the *p-value* of < 0.05.

### Predicted interaction network

The predicted protein–protein interaction (PPI) network among Eg-MAPK cascade genes was generated by STRING v11.5 software online (https://string-db.org/) based on an *B. mori* association model. The parameters were set as follows: meaning of network edges, confidence; minimum required interaction score, 0.7.

## Supplementary Information

**Additional file 1.**

## Data Availability

All data generated or analyzed during this study were included in this published article. The genome sequences of *E. grisescens* were downloaded from GenBank database (Accession No. PRJNA660825).

## References

[1] Stork NE (2018). How Many Species of Insects and Other Terrestrial Arthropods Are There on Earth?. Annu Rev Entomol.

[2] Deutsch CA (2018). Increase in crop losses to insect pests in a warming climate. Science.

[3] McLaren MR, Callahan BJ (1808). Pathogen resistance may be the principal evolutionary advantage provided by the microbiome. Philos Trans R Soc Lond B Biol Sci.

[4] Newton, K. and V.M. Dixit, Signaling in innate immunity and inflammation. Cold Spring Harb Perspect Biol, 2012. 4(3). 10.1101/cshperspect.a006049 PMC3282411 22296764

[5] Arthur JSC, Ley SC (2013). Mitogen-activated protein kinases in innate immunity. Nat Rev Immunol.

[6] Guo Z (2021). The regulation landscape of MAPK signaling cascade for thwarting Bacillus thuringiensis infection in an insect host. PLoS Pathog.

[7] Zhang W, Liu HT (2002). MAPK signal pathways in the regulation of cell proliferation in mammalian cells. Cell Res.

[8] Roux PP, Blenis J (2004). ERK and p38 MAPK-activated protein kinases: a family of protein kinases with diverse biological functions. Microbiol Mol Biol Rev.

[9] Widmann C (1999). Mitogen-activated protein kinase: conservation of a three-kinase module from yeast to human. Physiol Rev.

[10] Chang L, Karin M (2001). Mammalian MAP kinase signalling cascades. Nature.

[11] Krishna M, Narang H (2008). The complexity of mitogen-activated protein kinases (MAPKs) made simple. Cell Mol Life Sci.

[12] Shilo BZ (2014). The regulation and functions of MAPK pathways in Drosophila. Methods.

[13] Dong C, Davis RJ, Flavell RA (2002). MAP Kinases in the Immune Response. Annu Rev Immunol.

[14] Rasmussen MW (2012). MAP Kinase Cascades in Arabidopsis Innate Immunity. Front Plant Sci.

[15] Asai T (2002). MAP kinase signalling cascade in Arabidopsis innate immunity. Nature.

[16] Horton AA (2011). The mitogen-activated protein kinome from Anopheles gambiae: identification, phylogeny and functional characterization of the ERK, JNK and p38 MAP kinases. BMC Genomics.

[17] Ragab A (2011). Drosophila Ras/MAPK signalling regulates innate immune responses in immune and intestinal stem cells. EMBO J.

[18] Sakaguchi A, Matsumoto K, Hisamoto N (2004). Roles of MAP kinase cascades in Caenorhabditis elegans. J Biochem.

[19] Guo, Z., et al., MAPK-dependent hormonal signaling plasticity contributes to overcoming Bacillus thuringiensis toxin action in an insect host. Nature Communications, 2020. 11(1). 10.1038/s41467-020-16608-8 PMC7293236 32532972

[20] Li Z (2020). Toll signaling promotes JNK-dependent apoptosis in Drosophila. Cell Div.

[21] Myers AL (2018). Inflammatory production of reactive oxygen species by Drosophila hemocytes activates cellular immune defenses. Biochem Biophys Res Commun.

[22] Ip YT, Levine M (1994). Molecular genetics of Drosophila immunity. Curr Opin Genet Dev.

[23] Han ZS (1998). A conserved p38 mitogen-activated protein kinase pathway regulates Drosophila immunity gene expression. Mol Cell Biol.

[24] Dubovskiy IM (2013). Can insects develop resistance to insect pathogenic fungi?. PLoS ONE.

[25] Siegwart, M., et al., Resistance to bio-insecticides or how to enhance their sustainability: a review. Frontiers in Plant Science, 2015. 6. 10.3389/fpls.2015.00381 PMC4472983 26150820

[26] Berling M (2009). Cydia pomonella granulovirus genotypes overcome virus resistance in the codling moth and improve virus efficiency by selection against resistant hosts. Appl Environ Microbiol.

[27] Bravo A (2011). Bacillus thuringiensis: A story of a successful bioinsecticide. Insect Biochem Mol Biol.

[28] Trienens M, Rohlfs M (2011). Experimental evolution of defense against a competitive mold confers reduced sensitivity to fungal toxins but no increased resistance in Drosophila larvae. BMC Evol Biol.

[29] Han, J., J. Wu and J. Silke, An overview of mammalian p38 mitogen-activated protein kinases, central regulators of cell stress and receptor signaling. F1000Res, 2020. 9. 10.12688/f1000research.22092.1 PMC7324945 32612808

[30] Sluss HK (1996). A JNK signal transduction pathway that mediates morphogenesis and an immune response in Drosophila. Genes Dev.

[31] Kallio J (2005). Functional analysis of immune response genes in Drosophila identifies JNK pathway as a regulator of antimicrobial peptide gene expression in S2 cells. Microbes Infect.

[32] Wang S (2018). MKK4 from Litopenaeus vannamei is a regulator of p38 MAPK kinase and involved in anti-bacterial response. Dev Comp Immunol.

[33] Delaney JR (2006). Cooperative control of Drosophila immune responses by the JNK and NF-kappaB signaling pathways. EMBO J.

[34] Krishnaraj, T., et al., Identification of differentially expressed genes in dormant (banjhi) bud of tea (Camellia sinensis (L.) O. Kuntze) using subtractive hybridization approach. Plant Physiol Biochem, 2011. 49(6): p. 565–71. 10.1016/j.plaphy.2011.03.011 21481598

[35] Wei, C., et al., Draft genome sequence of Camellia sinensis var. sinensis provides insights into the evolution of the tea genome and tea quality. Proc Natl Acad Sci U S A, 2018. 115(18): p. E4151-E4158. 10.1073/pnas.1719622115 PMC5939082 29678829

[36] Drew L (2019). The growth of tea. Nature.

[37] Li ZQ (2019). Geographical Distribution of Ectropis grisescens (Lepidoptera: Geometridae) and Ectropis obliqua in China and Description of an Efficient Identification Method. J Econ Entomol.

[38] Pan Y (2021). Chromosome-level genome reference and genome editing of the tea geometrid. Mol Ecol Resour.

[39] Antony B, Sinu PA, Das S (2011). New record of nucleopolyhedroviruses in tea looper caterpillars in India. J Invertebr Pathol.

[40] Wang, Z., et al., Transcriptomic Analysis Reveals Insect Hormone Biosynthesis Pathway Involved in Desynchronized Development Phenomenon in Hybridized Sibling Species of Tea Geometrids (Ectropis grisescens and Ectropis obliqua). Insects, 2019. 10(11). 10.3390/insects10110381 PMC6920886 31683768

[41] Cao P (2018). Estimated assessment of cumulative dietary exposure to organophosphorus residues from tea infusion in China. Environ Health Prev Med.

[42] Sinha KK, Choudhary AK, Kumari P (2016). Chapter 15 - Entomopathogenic Fungi. Ecofriendly Pest Management for Food Security, Omkar, Omkar^Editors.

[43] Ortiz-Urquiza A, Keyhani NO (2013). Action on the Surface: Entomopathogenic Fungi versus the Insect Cuticle. Insects.

[44] Deshayes C (2017). Microbial Pest Control Agents: Are they a Specific And Safe Tool for Insect Pest Management?. Curr Med Chem.

[45] Yang, F., et al., Current status and prospect of entomopathogenic fungi for controlling insect and mite pests in tea plantations. Journal of Applied Entomology, 2022. n/a(n/a).

[46] Liu J (2021). In vitro transcriptomes analysis identifies some special genes involved in pathogenicity difference of the Beauveria bassiana against different insect hosts. Microb Pathog.

[47] Onsongo, S.K., et al., The Entomopathogenic Fungi Metarhizium anisopliae and Beauveria bassiana for Management of the Melon Fly Zeugodacus cucurbitae: Pathogenicity, Horizontal Transmission, and Compatability with Cuelure. Insects, 2022. 13(10). 10.3390/insects13100859 PMC9604353 36292807

[48] Long Y (2022). Analysis of the Humoral Immunal Response Transcriptome of Ectropis obliqua Infected by Beauveria bassiana. Insects.

[49] Manna PR, Stocco DM (2011). The role of specific mitogen-activated protein kinase signaling cascades in the regulation of steroidogenesis. J Signal Transduct.

[50] L'Allemain G (1994). Deciphering the MAP kinase pathway. Prog Growth Factor Res.

[51] Reiser V, Ammerer G, Ruis H (1999). Nucleocytoplasmic traffic of MAP kinases. Gene Expr.

[52] Li M, Liu J, Zhang C (2011). Evolutionary history of the vertebrate mitogen activated protein kinases family. PLoS ONE.

[53] Tena G (2001). Plant mitogen-activated protein kinase signaling cascades. Curr Opin Plant Biol.

[54] Cuevas, B.D., Mitogen-Activated Protein Kinase Kinase Kinases, in Encyclopedia of Cancer, M. Schwab, M. Schwab^Editors. 2017, Springer Berlin Heidelberg: Berlin, Heidelberg. p. 2872–2876.

[55] Hu, B., et al., GSDS 2.0: an upgraded gene feature visualization server. Bioinformatics, 2015. 31(8): p. 1296–7. 10.1093/bioinformatics/btu817 PMC4393523 25504850

[56] Gelfman S (2012). Changes in exon-intron structure during vertebrate evolution affect the splicing pattern of exons. Genome Res.

[57] Guo YJ (2020). ERK/MAPK signalling pathway and tumorigenesis. Exp Ther Med.

[58] Cargnello M, Roux PP (2011). Activation and function of the MAPKs and their substrates, the MAPK-activated protein kinases. Microbiol Mol Biol Rev.

[59] Lee, M.J. and M.B. Yaffe, Protein Regulation in Signal Transduction. Cold Spring Harb Perspect Biol, 2016. 8(6). 10.1101/cshperspect.a005918 PMC4888820 27252361

[60] Morrison, D.K., MAP kinase pathways. Cold Spring Harb Perspect Biol, 2012. 4(11). 10.1101/cshperspect.a011254 PMC3536342 23125017

[61] Pimienta G, Pascual J (2007). Canonical and alternative MAPK signaling. Cell Cycle.

[62] Zhou T (2004). Crystal structure of the TAO2 kinase domain: activation and specificity of a Ste20p MAP3K. Structure.

[63] Fang, C.Y., et al., The Diverse Roles of TAO Kinases in Health and Diseases. Int J Mol Sci, 2020. 21(20). 10.3390/ijms21207463 PMC7589832 33050415

[64] Champion A, Picaud A, Henry Y (2004). Reassessing the MAP3K and MAP4K relationships. Trends Plant Sci.

[65] Plotnikov A (2011). The MAPK cascades: signaling components, nuclear roles and mechanisms of nuclear translocation. Biochim Biophys Acta.

[66] Wei X (2020). The evolutionarily conserved MAPK/Erk signaling promotes ancestral T-cell immunity in fish via c-Myc-mediated glycolysis. J Biol Chem.

[67] Soares-Silva M (2016). The Mitogen-Activated Protein Kinase (MAPK) Pathway: Role in Immune Evasion by Trypanosomatids. Front Microbiol.

[68] Oliveros JC (2000). Expression profiles and biological function. Genome Inform Ser Workshop Genome Inform.

[69] Xu Y (2021). The Ras/MAPK pathway is required for regenerative growth of wing discs in the black cutworm Agrotis ypsilon. Insect Biochem Mol Biol.

[70] Dupre A, Haccard O, Jessus C (2011). Mos in the oocyte: how to use MAPK independently of growth factors and transcription to control meiotic divisions. J Signal Transduct.

[71] Fujiwara Y, Denlinger DL (2007). p38 MAPK is a likely component of the signal transduction pathway triggering rapid cold hardening in the flesh fly Sarcophaga crassipalpis. J Exp Biol.

[72] Huang, Z., et al., MAPK Signaling Pathway Is Essential for Female Reproductive Regulation in the Cabbage Beetle, Colaphellus bowringi. Cells, 2022. 11(10). 10.3390/cells11101602 PMC9140119 35626638

[73] Shvartsman SY, Coppey M, Berezhkovskii AM (2009). MAPK signaling in equations and embryos. Fly (Austin).

[74] Schaeffer HJ, Weber MJ (1999). Mitogen-activated protein kinases: specific messages from ubiquitous messengers. Mol Cell Biol.

[75] Raman M, Chen W, Cobb MH (2007). Differential regulation and properties of MAPKs. Oncogene.

[76] Tripolitsioti, D., M.A. Grotzer and M. Baumgartner, The Ser/Thr Kinase MAP4K4 Controls Pro-Metastatic Cell Functions. Journal of Carcinogenesis & Mutagenesis, 2017. 08(01).

[77] McCubrey JA (2007). Roles of the Raf/MEK/ERK pathway in cell growth, malignant transformation and drug resistance. Biochim Biophys Acta.

[78] McCubrey JA (2006). Roles of the RAF/MEK/ERK and PI3K/PTEN/AKT pathways in malignant transformation and drug resistance. Adv Enzyme Regul.

[79] Chen C (2020). TBtools: An Integrative Toolkit Developed for Interactive Analyses of Big Biological Data. Mol Plant.

[80] Wang Y (2012). MCScanX: a toolkit for detection and evolutionary analysis of gene synteny and collinearity. Nucleic Acids Res.

[81] Krzywinski M (2009). Circos: an information aesthetic for comparative genomics. Genome Res.

[82] Bailey, T.L., et al., MEME: discovering and analyzing DNA and protein sequence motifs. Nucleic Acids Res, 2006. 34(Web Server issue): p. W369–73. 10.1093/nar/gkl198 PMC1538909 16845028

[83] Blum M (2021). The InterPro protein families and domains database: 20 years on. Nucleic Acids Res.

[84] Sun R (2015). Genome-wide identification of auxin response factor (ARF) genes and its tissue-specific prominent expression in Gossypium raimondii. Funct Integr Genomics.

