# Correction to: Genome-wide identifcation and immune response analysis of mitogen-activated protein kinase cascades in tea geometrid, *Ectropis grisescens* Warren (Geometridae, Lepidoptera)

**DOI:** 10.1186/s12864-023-09508-w

**Published:** 2023-07-13

**Authors:** Xiaozhu Wu, Chenghua Zhou, Xiaofang Li, Jingyi Lin, Luis Carlos Ramos Aguila, Feng Wen, Liande Wang

**Affiliations:** 1 State Key Laboratory of Ecological Pest Control for Fujian and Taiwan Crops, Key Laboratory of Biopesticides and Chemical Biology, Ministry of Education, College of Plant Protection, Fujian Agriculture and Forestry University, Fuzhou, 350002 China; 2 School of Pharmacy and Life Science, Jiujiang University, Jiujiang, 332000 China; 3 School of Biological Science and Food Engineering, Chuzhou University, Chuzhou, 239099 China

**Correction to**: ***BMC Genomics *24, 344 (2023)**

10.1186/s12864-023-09446-7

Following publication of the original article [1], it was reported that there was an error in the affiliation numbering. The correct affiliations are given in this Correction and the original article has been updated.
